# *TET2* germline mutation in a patient with sequential lymphoid malignancies: a novel case report

**DOI:** 10.1007/s00277-026-06930-4

**Published:** 2026-03-17

**Authors:** Xia Mao, Kefeng Shen, Jin Wang, Zhiqiong Wang, Qilin Ao, Chunyan Wang, Min Xiao

**Affiliations:** 1 Department of Hematology, Tongji Hospital, Tongji Medical College, Huazhong University of Science and Technology, 1095 Jiefang Avenue, Qiaokou District, Wuhan, 430030 China; 2 Institute of Pathology, Tongji Hospital, Tongji Medical College, Huazhong University of Science and Technology, Wuhan, China; 3 Key Laboratory of Vascular Aging, Ministry of Education, Tongji Hospital, Tongji Medical College, Huazhong University of Science and Technology, 1095 Jiefang Avenue, Wuhan, 430030 China

**Keywords:** TET2 germline mutation, Sequential lymphoid malignancies, Mixed cellularity classical hodgkin lymphoma, Angioimmunoblastic T-cell lymphoma, T-cell acute lymphoblastic leukemia

## Abstract

We report a case of a 30-year-old male patient with a germline heterozygous *TET2* R1354fs mutation, who successively developed three distinct lymphoid malignancies: mixed cellularity classical Hodgkin lymphoma (MCCHL), angioimmunoblastic T-cell lymphoma (AITL), and T-cell acute lymphoblastic leukemia (T-ALL). Sequencing analysis of the oral mucosa and bone marrow specimens during the remission phase confirmed the germline origin of the *TET2* mutation. This represents the first documented case of the sequential transformation across three distinct lymphoid malignancies attributable to a germline *TET2* mutation, underscoring its pivotal role in lymphomagenesis and clonal evolution.

**Supplementary Information:**

The online version contains supplementary material available at 10.1007/s00277-026-06930-4.

## Introduction

*TET2*, located on chromosome 4q24, is a tumor suppressor gene that encodes a key member of the TET dioxygenase family, which includes *TET1*, *TET2*, and *TET3* [1]. This enzyme plays critical regulatory roles in hematopoietic processes, including hematopoietic stem cell (HSC) self-renewal, lineage commitment, and terminal monocytic differentiation [1–2]. Preclinical studies using *TET2*-deficient murine models demonstrate that loss-of-function mutations result in myeloproliferative phenotypes, which are characterized by bone marrow dysplasia, splenomegaly, monocytosis, and lymphomagenesis [3–6]. *TET2* deficiency is known to promote the pathological expansion of multipotent progenitors and myeloid precursors, leading to the accumulation of premalignant clones [3–6]. While somatic mutations of *TET2* are well-documented in hematological malignancies, germline mutations in *TET2* remain exceptionally rare. This study reports the first documented case of a patient with a *TET2* germline mutation who developed sequential lymphoid neoplasms, thereby enhancing our understanding of *TET2*’s role in tumorigenesis across hematopoietic lineages and offering valuable insights into germline predisposition mechanisms in lymphomagenesis.

## Case presentation

A 30-year-old man presented with a 4-month history of a painless left cervical mass; he had no B symptoms. The complete blood count at onset was normal, and there was no evidence of immune thrombocytopenia. Physical examination revealed no hepatosplenomegaly, fever, or history of recurrent infection. Left cervical lymph-node biopsy showed architectural destruction with Hodgkin–Reed–Sternberg (HRS) cells (CD30+++/CD15+/PAX-5(dim+)/CD20(dim+)/LCA−/EBV+ (by in situ hybridization for Epstein–Barr virus encoded RNA (EBER))) against a background of abundant CD3 + small T cells with regressed follicular dendritic-cell meshworks. T-cell receptor gene rearrangement studies confirmed a polyclonal profile, supporting a diagnosis of mixed cellularity classical Hodgkin lymphoma (MCCHL) (Supplementary Figure S1-A). Imaging examination (CT/MRI) suggested stage IIE (Ann Arbor). The patient received 4 cycles of ABVD chemotherapy (doxorubicin 48 mg on days 1/15, bleomycin 19 mg on days 1/15, vinblastine 12 mg on days 1/15, dacarbazine 700 mg on days 1/15), followed by neck/axillary radiotherapy (30.6 Gy/17 fractions). The chemotherapy dose for the 5th and 6th cycles was adjusted to 80% due to radiotherapy toxicity. After 6 cycles of chemotherapy, the patient achieved complete remission. Follow-up examinations at regular intervals revealed no signs of recurrence. Six years later (at 36 years old), the patient developed bilateral inguinal masses. The second biopsy revealed effaced nodal architecture with residual atrophic follicles and paracortical infiltrates of neoplastic cells associated with hyperplastic high-endothelial venules. The tumor cells were focally distributed and exhibited a medium-sized morphology with pale cytoplasm. Immunohistochemical analysis showed that strong positivity for PD-1, along with expression of CD3 and CD4, while CD10 and BCL6 show scattered positivity. Occasional Hodgkin-like large cells were CD30+, MUM-1+, PAX-5(dim+), LCA−, CD15−, CD20−, and EBER+. These findings established a diagnosis of angioimmunoblastic T-cell lymphoma (AITL) (Supplementary Figure S1-B). Flow cytometry confirmed clonal T-cell proliferation with the immunophenotype CD3−, CD4+, CD7−, CD5+, PD-1(bright+), CD200+, CD10−, CD30−, TRBC1+ (Supplementary Figure S2). The patient was treated with 6 cycles of DVCP + cidazolam regimen (cyclophosphamide 1500 mg on day 1, pirarubicin 100 mg on day 1, vindesine 4 mg on day 1, cidazolam 30 mg on days 1/4/8/11, dexamethasone 15 mg on days 1–5). Four years after the AITL diagnosis (at 41 years old), the patient visited the hospital due to cough, sputum, rhinorrhea, and general fatigue. Imaging showed generalized lymphadenopathy with a huge mediastinal mass (encasing large blood vessels and trachea) and serous cavity effusion. Routine blood count showed platelet count was 55 × 10^⁹/^L with normal WBC and Hb counts. Cytological examination of pleural effusion demonstrated malignant lymphocytes, and flow cytometric analysis identified a population of immature T-lymphoblasts. Bone marrow (BM) morphology showed 99% lymphoblasts, which were identified as T-lymphoblasts by immunohistochemistry and flow cytometry (Supplementary Figure S3). The immunophenotype was characterized by CD45dim expression, cytoplasmic CD3+, CD7(bright+), CD4+, CD8(dim+), CD1a+, CD99+, and TdT+, with monoclonal expression of cytoplasmic TRBC1. Flow cytometry did not detect neoplastic mature T cells. Immunohistochemistry (IHC) performed on BM biopsy samples showed no CD30-positive Reed-Sternberg (RS) cells. Due to failure of conventional karyotyping culture for the bone marrow sample, copy number variation (CNV, genome-wide shallow-depth sequencing) analysis was performed as a supplementary test; this revealed 46, XY, del(5)(p15.33p15.32).

**Fig. 1:**
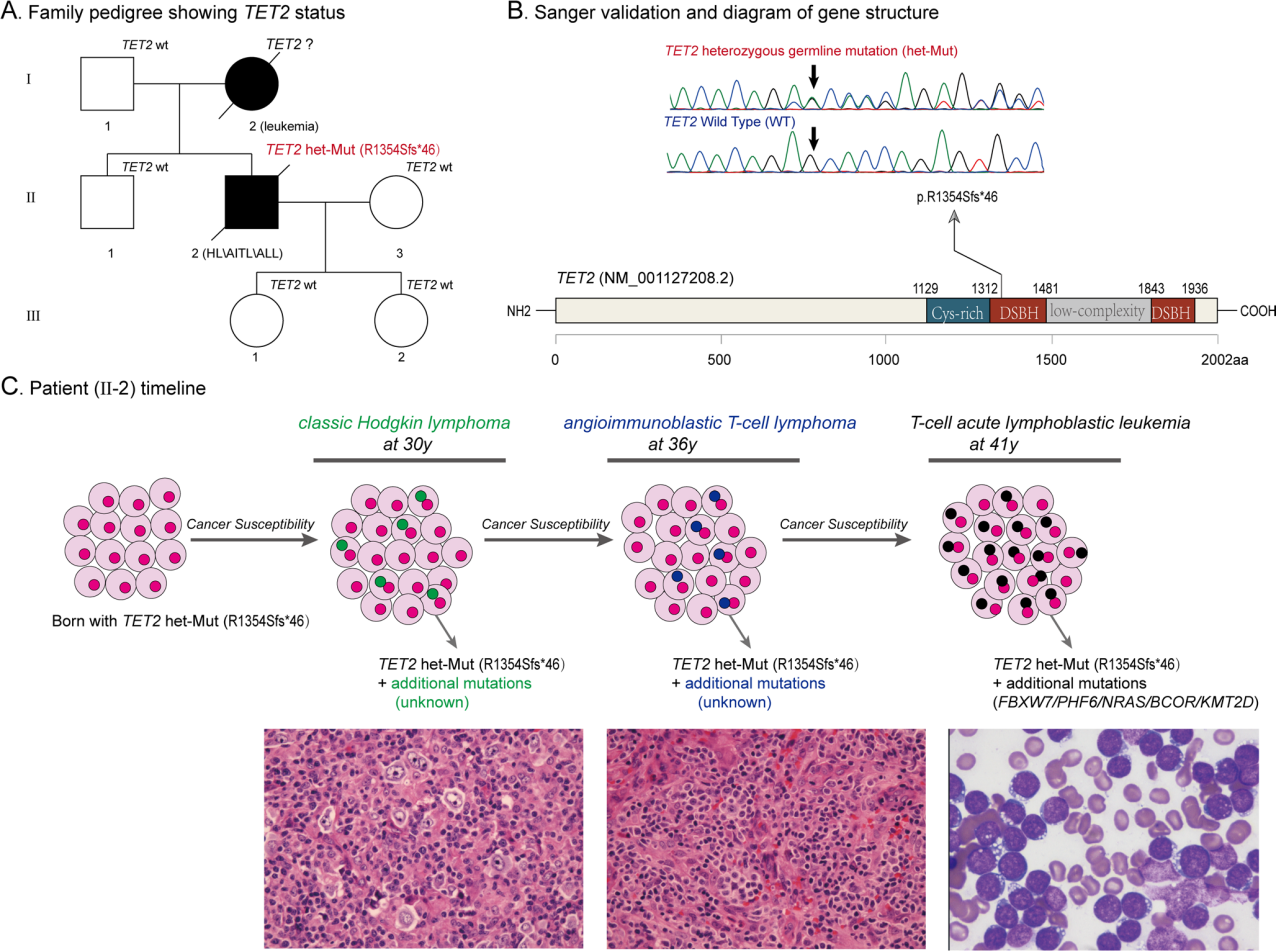
A brief summary of the medical history and comprehensive molecular-genetic analysis. (**A**) Family pedigree showing *TET2* mutation status. Affected individuals with hematological malignancies are shaded in black. HL Hodgkin lymphoma, AITL angioimmunoblastic T - cell lymphoma, ALL acute lymphoblastic leukemia. (**B**) Sanger sequencing with buccal swab samples showing the c.4062_4063del (p.R1354fs) mutation in the *TET2* gene (NM_001127208.2). (**C**) Patient timeline of key clinical events and laboratory test results. The patient harbored a congenital germline heterozygous *TET2* mutation that conferred genomic instability and tumor predisposition, driving a multistep oncogenic evolution.The neoplastic progression sequentially manifested as MCCHL, then progressed to AITL, and ultimately culminated in T-ALL.

To elucidate the molecular mechanisms, comprehensive genetic testing was performed on formalin-fixed paraffin-embedded (FFPE) tissue samples from the initial diagnosis of MCCHL, the secondary diagnosis of AITL, and BM aspirate samples from the final diagnosis of T-cell acute lymphoblastic leukemia (T-ALL). Targeted next-generation sequencing (NGS) of the initial MCCHL identified a *TET2* (R1354fs: 39.56%) mutation. Subsequent NGS of the secondary AITL FFPE sample revealed *TET2* mutations (R1354fs: 38.89%, S462fs: 11.64%). NGS of the final T-ALL BM aspirate sample demonstrated *TET2* (R1354fs: 97.80%), *BCOR* (L261fs: 97.36%), *NRAS* (G12D: 48.21%), *PHF6* (E139*: 96.11%), *FBXW7* (R465H: 97.02%), *EZH2* (L128S: 48.21%), and *KMT2D* (K3001fs: 47.36%) mutations. RNA sequencing (RNA-seq) detected the *TET2* (R1354fs) frameshift deletion with a variant allele frequency of 24%, while no specific fusion genes were identified. Comparative analysis of the three NGS datasets revealed consistent *TET2* (R1354fs) mutations across all tumor samples with high variant allele frequencies (39.56%, 38.89%, and 97.80%, respectively). Notably, the family history indicated maternal death from acute leukemia 26 years prior (though diagnostic details were limited and DNA unavailable for sequencing), prompting suspicion of a heritable *TET2* mutation. Sanger sequencing of buccal swab samples confirmed the heterozygous germline status of the mutation (Fig. 1). Familial segregation analysis revealed that the proband’s father, younger brother, and two daughters were *TET2* mutation negative. Whole Exome Sequencing (WES) of remission-phase BM samples further validated this finding, confirming a heterozygous germline *TET2* mutation.

The patient was eventually diagnosed with T-ALL and received the first cycle of intensive chemotherapy, which included cyclophosphamide (1430 mg on days 1 and 22), pirarubicin (76 mg on days 1–3 and 22–24), vindesine (4 mg weekly), bortezomib (2.5 mg on days 2, 9, 16, and 22), and dexamethasone (15 mg on days 1–14). During this period of intensive chemotherapy, the patient developed a pulmonary fungal infection. Ten weeks following the initial cycle, a second-line treatment regimen was commenced, consisting of cyclophosphamide (750 mg/m² on days 1 and 8), cytarabine (100 mg/m² on days 1–7), and etoposide (100 mg on days 1–5). One month later, an intrathecal injection of methotrexate, cytarabine, and dexamethasone was administered. Subsequently, the intensive treatment was adjusted to mitoxantrone 8 mg/m² on days 1–3 combined with cytarabine 150 mg/m² on days 1–7, supplemented with anti-infection and supportive care. After the last VDCLP chemotherapy regimen, the patient developed severe BM suppression, followed by pulmonary aspergillosis, multidrug-resistant bacteremia (Stenotrophomonas maltophilia), and type I respiratory failure. Despite active resuscitation efforts, the patient eventually died of septic shock complicated by severe pneumonia.

## Discussion

We report a rare case of a 30-year-old male patient harboring a heterozygous germline *TET2* mutation, whose disease trajectory exhibited a sequential progression from MCCHL to AITL and ultimately to T-ALL/LBL.

The core controversy in this case revolves around the pathological characteristics at the initial diagnosis: despite the marked differences observed between the initial diagnosis and the histological features during disease progression, several critical considerations must be addressed. Firstly, in terms of pathological differential diagnosis, AITL and MCCHL have overlapping features in conventional histology [7–9]. However, in this case, the initial biopsy demonstrated a polyclonal pattern of T-cell receptor (TCR) gene rearrangement. Moreover, retrospective next-generation sequencing (NGS) performed on the cHL specimen did not identify AITL specific mutations (i.e. *RHOA* G17V, *IDH2*). Taken together, these molecular findings are more consistent with a diagnosis of classical Hodgkin lymphoma. Secondly, there is a contradiction in epidemiological characteristics: AITL is more common in the elderly population (median age of onset 65–68 years, 60%-75% of cases ≥ 60 years [10]), while the peak incidence of cHL is between 30 and 40 years old. The patient’s age at initial diagnosis (30 years old) is more consistent with the typical distribution of MCCHL. Observing the treatment response, the patient’s initial complete remission to the ABVD regimen (doxorubicin + bleomycin + vinblastine + dacarbazine) (lasting for more than 5 years) is in line with the typical treatment response pattern of classical Hodgkin lymphoma (cHL). In contrast, the remission rate of AITL patients to the CHOP regimen (cyclophosphamide + doxorubicin + vincristine + prednisone) is usually < 50%, and the median progression-free survival is only 8-12 months [11]. These characteristics are consistent with the continuous failure of complete remission and rapid recurrence and progression of patients after late treatment.

The dynamics of *TET2* variant allele frequency (VAF) in this patient, ranging from 38% in germline heterozygous mutations to 97% in T-ALL/LBL stage, suggest potential molecular evolutionary events. The mechanisms that can explain this phenomenon mainly involve three aspects: First, loss of heterozygosity (LOH), a classic pathway for biallelic inactivation, may be achieved through two molecular forms: (1) Physical deletion of the 4q24 region (where the *TET2* gene is located), leading to the loss of the wild-type allele and resulting in a functionally homozygous mutant state; (2) Formation of uniparental disomy through gene conversion or homologous recombination events, which retains the diploid chromosome but makes both alleles mutant [12, 13]. Notably, although CNV analysis detected a deletion at 5p15.33p15.32 and revealed no large-scale deletions on chromosome 4, it does not completely exclude the possibility of local LOH. This is because conventional CNV detection techniques (with a resolution typically > 5 Mb) may miss microdeletions (< 5 Mb) or recombination events leading to region-specific LOH in the 4q24 area. Second, the mechanism of somatic second mutation needs to be considered. Hematopoietic stem cells originally carrying germline heterozygous mutations may acquire another mutation in the other allele (including point mutations, frameshift mutations, or splice site mutations) during the process of tumor transformation, leading to biallelic inactivation. This mechanism can directly increase the theoretical VAF from 38% to 97% without accompanying copy number changes [14]. Third, the role of clonal selection pressure in the evolution of mutation abundance cannot be ignored. Malignant clones with biallelic inactivation of *TET2* may gain a proliferative advantage (such as enhanced self-renewal capacity of stem cells), escape immune surveillance, or acquire treatment resistance, and thus gradually become the dominant clone in tumor evolution [15]. To distinguish the above mechanisms, high-density SNP array (with a resolution of 50 kb) is required to precisely locate the 4q24 region, detect microdeletions or copy-neutral LOH, or apply third-generation sequencing technology (such as PacBio HiFi) for long-read sequencing to clarify the spatial distribution of biallelic mutations and recombination events, to further precisely parse the molecular driving mechanisms of increased *TET2* mutation abundance.

The occurrence of hematological malignancies is typically driven by somatic mutations, but recent studies suggest that hereditary tumor susceptibility may be more important than previously thought. Germline single or double allele mutations in the human *TET2* gene are associated with susceptibility to malignancies [16, 17]. E. Kaasinen et al. reported a Finnish family carrying a heterozygous germline *TET2* frameshift mutation, with multiple members suffering from lymphoma [18]; N. Duployez et al. further identified *TET2* germline frameshift mutations in French families, leading to three siblings developing myeloid malignancies [17]. In addition, J. Spegarova et al. first reported autosomal recessive *TET2* germline double allele defects, manifested as severe immune deficiency and autoimmune lymphoproliferative syndrome (ALPS), significantly increasing the risk of lymphoma [16]; M. López-Nevado et al. also found two patients carrying *TET2* single or double allele mutations, both presenting with ALPS-like phenotypes [19]. Harrop et al. describes four unrelated patients with heterozygous germline *TET2* loss-of-function variants who developed nodular lymphocyte predominant Hodgkin Lymphoma (NLPHL) or related B-cell lymphomas on a background of chronic lymphadenopathy and autoimmune features, establishing that monoallelic germline *TET2* deficiency can recapitulate an ALPS-like predisposition syndrome [20]. In contrast to previous reports of ALPS-associated lymphomas, we identified, for the first time, a patient with a heterozygous germline *TET2* mutation whose disease course followed a multistep evolution: from mixed cellularity classical Hodgkin lymphoma (MCCHL) to mature T-cell lymphoma, and ultimately to acute T-lymphoblastic leukemia (T-ALL). The patient sequentially developed three distinct lymphoid neoplasms without exhibiting ALPS-related features.

*TET2* mutations are well-known precancerous lesions, as *TET2* haploinsufficiency requires synergistic interactions with additional genetic alterations to malignant transformation. However, in this case, typical secondary driver mutations were not identified through whole-exome sequencing and RNA-Seq analysis, suggesting the potential involvement of other undetected molecular abnormalities or alternative mechanisms contributing to tumorigenesis. The *TET2* gene plays a key role in maintaining genomic stability, and its mutations can lead to aberrant DNA methylation, thereby disrupting gene expression and cellular function, inducing genomic instability, and ultimately promoting lymphomagenesis. In addition to *TET2* mutations, it may also involve alterations in other genes, epigenetic factors, immune factors, clonal evolution, and other environmental and infectious factors. These factors may interact with each other to jointly promote the occurrence and development of lymphoma. To further elucidate the etiology in this patient, more comprehensive genetic profiling and epigenetic analysis are warranted to uncover the underlying molecular mechanisms of the disease.

In conclusion, germline *TET2* mutations significantly contribute to the pathogenesis of hematological malignancies. This case not only broadens the phenotypic spectrum associated with *TET2* germline mutations but also reveals the continuous influence of genetic predisposition in the dynamic evolution of tumors. This provides a molecular basis for early surveillance in high-risk populations and offers novel insights into diagnostic and therapeutic strategies for the disease.

## Supplementary Information

Below is the link to the electronic supplementary material.

Supplementary Material 1

Supplementary Figure 1 (PNG 9.46 MB)

High Resolution Image (TIF 8.33 MB)

Supplementary Figure 2 (PNG 455 KB)

High Resolution Image (TIF 17.5 MB)

Supplementary Figure 3 (PNG 2.03 MB)

High Resolution Image (TIF 40.1 MB)

## Data Availability

No datasets were generated or analysed during the current study.
