# Supplementary material for: TET2 germline mutation in a patient with sequential lymphoid malignancies: a novel case report
Source: Ann Hematol. 2026 Mar 17;105(4):184. doi: 10.1007/s00277-026-06930-4 (PMC12995931; doi:10.1007/s00277-026-06930-4)
Supplement: Supplementary file 1 — Supplementary Material 1 [file 277_2026_6930_MOESM1_ESM.docx]

**Supplementary Material**

Targeted next-generation sequencing (NGS) was performed on the initial formalin-fixed paraffin-embedded (FFPE) tissue samples obtained at the first diagnosis of MCCHL, the subsequent diagnosis of AITL, as well as on the bone marrow (BM) aspirate collected at the final diagnosis of T-ALL. A custom panel covering 521 genes recurrently mutated in hematologic malignancies was employed, achieving a mean sequencing depth greater than 2000× and an analytical sensitivity of approximately 1%. Library preparation was conducted using the AmpliSeq™ Library PLUS kit for Illumina, and paired-end sequencing was performed on the NextSeq™ 550 platform (Illumina, San Diego, USA). Sequence alignment and variant calling were carried out using the DNA Amplicon workflow with default parameters on the Illumina BaseSpace Sequence Hub. All detected variants were subsequently annotated and interpreted with ANNOVAR using standard filtering and functional annotation pipelines.

For whole-exome sequencing (WES) of remission-phase BM samples from the T-ALL stage, libraries were generated using the Fast Library Prep Kit, and exonic regions were enriched with the AIExome Enrichment Kit V1 (iGeneTech, Beijing, China). Sequencing was performed on an Illumina platform using 150-bp paired-end reads. Raw reads underwent quality assessment and filtering with FastQC prior to alignment to the GRCh37 reference genome using BWA. After removal of duplicate reads, single-nucleotide variants (SNVs) and small insertions/deletions (indels) were called and annotated using the GATK pipeline.

To identify potential fusion genes, RNA sequencing was performed on BM samples collected at the final diagnosis of T-ALL using an Illumina sequencing platform. Fusion transcripts were detected using STAR-Fusion (STAR Methods; https://github.com/alexdobin/STAR).

**Table S1. Targeted 521-gene sequencing panel**

| *ABL1* | *FANCG* | *PIGA* | *TRAF3* | *CTCF* | *HIST1H2BK* | *NUDT15* | *SOCS1* |
| --- | --- | --- | --- | --- | --- | --- | --- |
| *ACD* | *FANCI* | *PIK3CD* | *TSR2* | *CTLA4* | *HIST1H3B* | *OR10A2* | *SOD2* |
| *ANKRD26* | *FANCL* | *PIM1* | *U2AF1* | *CTNNB1* | *HIST1H3D* | *P2RY8* | *SOS1* |
| *ARC* | *FANCM* | *PLCG1* | *UBE2T* | *CUX1* | *HIST1H3H* | *PBRM1* | *SPEN* |
| *ARID1A* | *FAS* | *PLCG2* | *VAV1* | *CXCR5* | *HIST1H3J* | *PCBP1* | *SPI1* |
| *ARID1B* | *FBXW7* | *POT1* | *VHL* | *CYBA* | *HIST1H4E* | *PDCD1* | *SRCAP* |
| *ARID2* | *FGFR3* | *PPM1D* | *WAS* | *CYP2B6* | *HIST1H4J* | *PDE4DIP* | *SRP54* |
| *ASXL1* | *FLT3* | *PRDM1* | *WRAP53* | *CYP2C19* | *HIST2H2BE* | *PDGFRA* | *SRSF1* |
| *ASXL2* | *FOXO1* | *PRKCB* | *WT1* | *CYP2C8* | *HLA-DRB1* | *PDS5B* | *STAG1* |
| *ASXL3* | *FYN* | *PRPF8* | *XRCC2* | *CYP3A4* | *HNRNPU* | *PIK3CA* | *STAT5A* |
| *ATM* | *G6PC3* | *PTEN* | *ZBTB7A* | *CYP3A5* | *HVCN1* | *PIK3R1* | *STAT6* |
| *B2M* | *GATA1* | *PTPN1* | *ZEB2* | *DARS* | *IFNL3* | *PLXNB3* | *STIM1* |
| *BCL2* | *GATA2* | *PTPN11* | *ZRSR2* | *DCAF6* | *IGLL5* | *PML* | *STX11* |
| *BCL6* | *GATA3* | *RAD21* | *ABCA13* | *DCDC1* | *IL13RA1* | *PMS2* | *STXBP2* |
| *BCOR* | *GFI1* | *RAD51* | *ABCB1* | *DCTD* | *IL1RAPL2* | *PNPLA3* | *SUZ12* |
| *BCORL1* | *GNA13* | *RAD51C* | *ABCC2* | *DDX10* | *IL4R* | *POSTN* | *TAF1* |
| *BIRC3* | *GNAI2* | *RAF1* | *ABCG2* | *DDX11* | *IL6* | *POU2AF1* | *TBL1XR1* |
| *BLM* | *HAX1* | *RB1* | *ACTB* | *DHX30* | *IMPDH2* | *POU2F2* | *TCL1A* |
| *BLNK* | *HNRNPA2B1* | *RHOA* | *ACTG1* | *DHX58* | *IRF2BP2* | *PPFIA2* | *TCTN2* |
| *BRAF* | *ID3* | *RPL11* | *ADD2* | *DIS3* | *IRF4* | *PRF1* | *TFAP4* |
| *BRCA1* | *IDH1* | *RPL15* | *ADGRV1* | *DNM2* | *IRF8* | *PROX1-AS1* | *TLR2* |
| *BRCA2* | *IDH2* | *RPL23* | *ADSL* | *DNMT1* | *IRX2* | *PRPS1* | *TMEM30A* |
| *BRIP1* | *IKZF1* | *RPL26* | *AKAP6* | *DOK5* | *ITPA* | *PRUNE2* | *TMSB4X* |
| *BTK* | *IKZF2* | *RPL27* | *AKT3* | *DROSHA* | *ITPR3* | *PTPN6* | *TNF* |
| *CALR* | *IKZF3* | *RPL31* | *ALK* | *DTX1* | *JARID2* | *PTPRC* | *TNFSF14* |
| *CARD11* | *IL7R* | *RPL35A* | *AOC2* | *DYNC2H1* | *KDM5C* | *PTPRD* | *TOX* |
| *CBL* | *INO80* | *RPL5* | *ARID3A* | *EBF1* | *KIAA1671* | *PTPRM* | *TPMT* |
| *CCND1* | *INPP5D* | *RPS10* | *ATP6AP1* | *ECT2L* | *KLHL14* | *PTPRN2* | *TRAF2* |
| *CCR4* | *ITPKB* | *RPS17* | *ATP6V1B2* | *EED* | *KLHL6* | *RAB27A* | *TRRAP* |
| *CCR7* | *JAK1* | *RPS19* | *ATRX* | *EEF1A1* | *KMT2B* | *RARA* | *TSPAN19* |
| *CD28* | *JAK2* | *RPS24* | *BACH2* | *EGR1* | *LEF1* | *RBBP6* | *TTN* |
| *CD58* | *JAK3* | *RPS26* | *BCL11A* | *EGR2* | *LINC00251* | *REL* | *TYK2* |
| *CD79A* | *JUNB* | *RPS27* | *BCL11B* | *EIF4A1* | *LRRN3* | *RELN* | *U2AF2* |
| *CD79B* | *KANSL1* | *RPS28* | *BCL7A* | *ERBB3* | *LTB* | *RFX7* | *UBE2A* |
| *CDKN1B* | *KDM6A* | *RPS29* | *BCR* | *ERBB4* | *LUC7L2* | *RGS1* | *UGT1A1* |
| *CDKN2A* | *KIT* | *RPS7* | *BIRC6* | *ERCC1* | *LYN* | *RIT1* | *UGT1A8* |
| *CDKN2B* | *KLF2* | *RRAGC* | *BMP7* | *ERG* | *LYST* | *ROBO1* | *UNC13D* |
| *CEBPA* | *KMT2A* | *RTEL1* | *BRCC3* | *FAM46C* | *MAGEC2* | *ROBO2* | *USB1* |
| *CHD8* | *KMT2C* | *RUNX1* | *BRINP3* | *FAT1* | *MAGT1* | *RP1L1* | *USH2A* |
| *CREBBP* | *KMT2D* | *SAMD9* | *BTG1* | *FAT4* | *MAP3K14* | *RPL10* | *USP7* |
| *CSF1R* | *KRAS* | *SAMD9L* | *BTG2* | *FBXO11* | *MED12* | *RPL18* | *VMA21* |
| *CSF3R* | *MAP2K1* | *SBDS* | *CARMIL2* | *FCGR3A* | *MGA* | *RPS15* | *VMP1* |
| *CTC1* | *MAPK1* | *SETBP1* | *CCL4* | *FGA* | *MME* | *RRM1* | *WDFY3* |
| *CXCR4* | *MBD4* | *SF3B1* | *CCND2* | *FGB* | *MPEG1* | *RRM2* | *XIAP* |
| *DDX3X* | *MECOM* | *SGK1* | *CCND3* | *FGFR1* | *MS4A1* | *RRM2B* | *XPO1* |
| *DDX41* | *MEF2B* | *SH2B3* | *CD274* | *FGG* | *MSH2* | *S1PR2* | *XRCC5* |
| *DHX15* | *MET* | *SH2D1A* | *CD70* | *FPGT* | *MTHFR* | *SAMHD1* | *YLPM1* |
| *DKC1* | *MFHAS1* | *SLX4* | *CD83* | *FZD3* | *MTOR* | *SCG3* | *YTHDF2* |
| *DNAJC21* | *MLH1* | *SMARCA4* | *CDA* | *GCSAM* | *MTRR* | *SERPINB3* | *YY1* |
| *DNMT3A* | *MPL* | *SMC1A* | *CDC25C* | *GDF11* | *MYB* | *SERPINE1* | *ZC3H12A* |
| *DUSP2* | *MSH6* | *SMC3* | *CDKN2C* | *GNAS* | *MYC* | *SETD1A* | *ZCCHC11* |
| *EFL1* | *MYBBP1A* | *SRP72* | *CEP72* | *GNB1* | *NCOR1* | *SETD1B* | *ZFP36L1* |
| *ELANE* | *MYD88* | *SRSF2* | *CHD2* | *GPR37* | *NCOR2* | *SETD2* | *ZFP36L2* |
| *EP300* | *NAF1* | *STAG2* | *CIITA* | *GRB2* | *NFATC2* | *SETD5* | *ZMYM3* |
| *EPHA7* | *NF1* | *STAT3* | *CISH* | *GRM7* | *NFKBIA* | *SETDB2* | *ZNF217* |
| *EPOR* | *NHP2* | *STAT5B* | *CLGN* | *GSTM1* | *NFKBIE* | *SF1* | *ZNF292* |
| *ERCC4* | *NOP10* | *TCF3* | *CNKSR2* | *GSTP1* | *NLRC4* | *SGPP1* | *ZNF423* |
| *ETNK1* | *NOTCH1* | *TERC* | *CNOT3* | *GTF2I* | *NLRP8* | *SIN3A* | *ZNF608* |
| *ETV6* | *NOTCH2* | *TERT* | *COL6A3* | *HDAC9* | *NOL9* | *SLC22A1* | *ZNF80* |
| *EZH2* | *NPM1* | *TET2* | *COQ7* | *HIST1H1B* | *NOTCH3* | *SLC29A1* |  |
| *FANCA* | *NRAS* | *TET3* | *CPA2* | *HIST1H1C* | *NOTCH4* | *SLCO1A2* |  |
| *FANCB* | *PALB2* | *TINF2* | *CRBN* | *HIST1H1D* | *NR3C1* | *SLCO1B1* |  |
| *FANCC* | *PARN* | *TNFAIP3* | *CRIP1* | *HIST1H1E* | *NRXN3* | *SLITRK3* |  |
| *FANCD2* | *PAX5* | *TNFRSF14* | *CRLF2* | *HIST1H2AC* | *NSD2* | *SMAD4* |  |
| *FANCE* | *PDGFRB* | *TNFRSF1B* | *CSF2RB* | *HIST1H2AM* | *NT5C2* | *SMARCB1* |  |
| *FANCF* | *PHF6* | *TP53* | *CSNK1A1* | *HIST1H2BC* | *NTRK1* | *SNTB2* |  |
