# Supplementary figures and images for: TET2 germline mutation in a patient with sequential lymphoid malignancies: a novel case report
Source: Ann Hematol. 2026 Mar 17;105(4):184. doi: 10.1007/s00277-026-06930-4 (PMC12995931; doi:10.1007/s00277-026-06930-4)

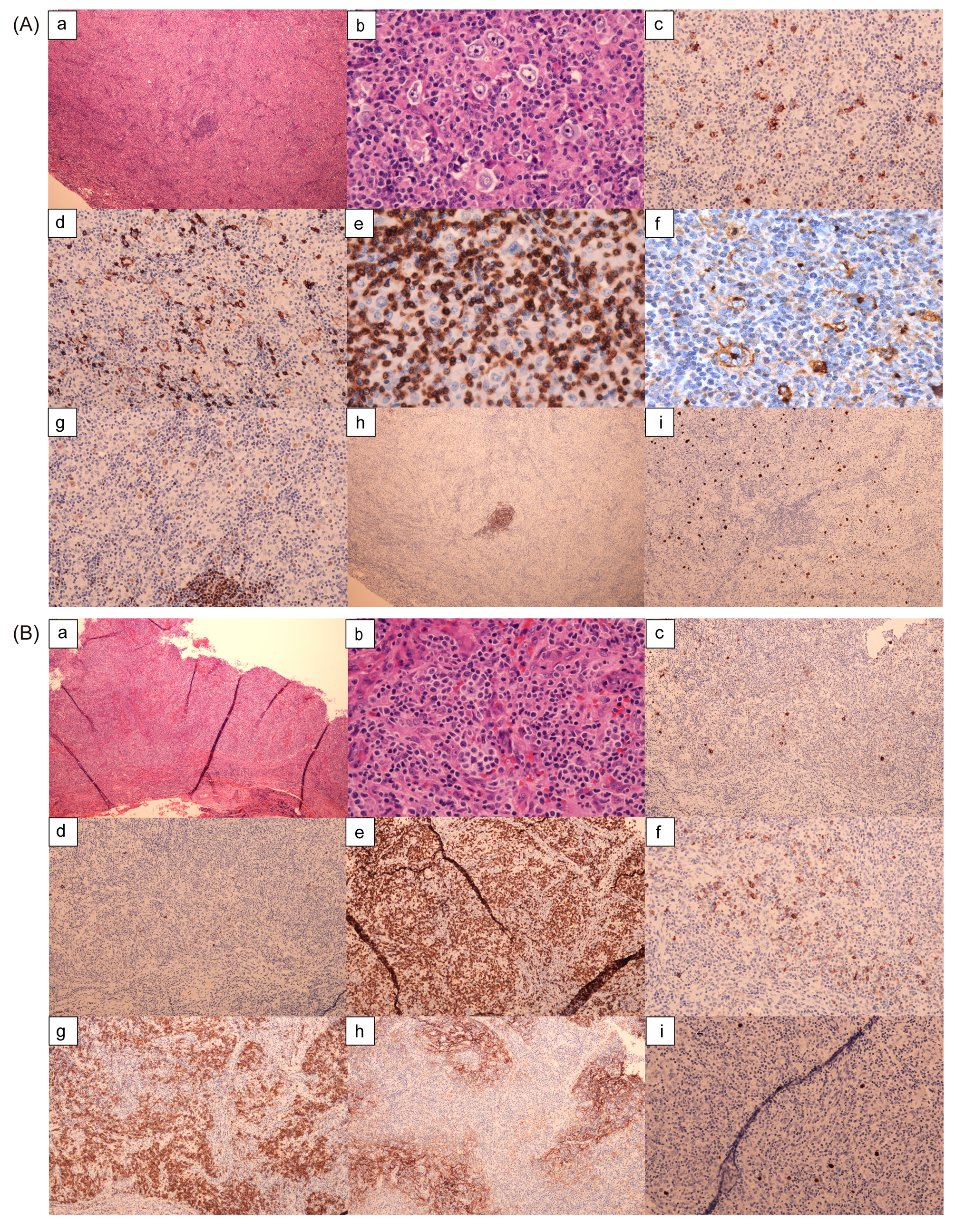

Supplement: Supplementary file 2 — Supplementary Figure 1 (PNG 9.46 MB) [file 277_2026_6930_Fig2_ESM.png]

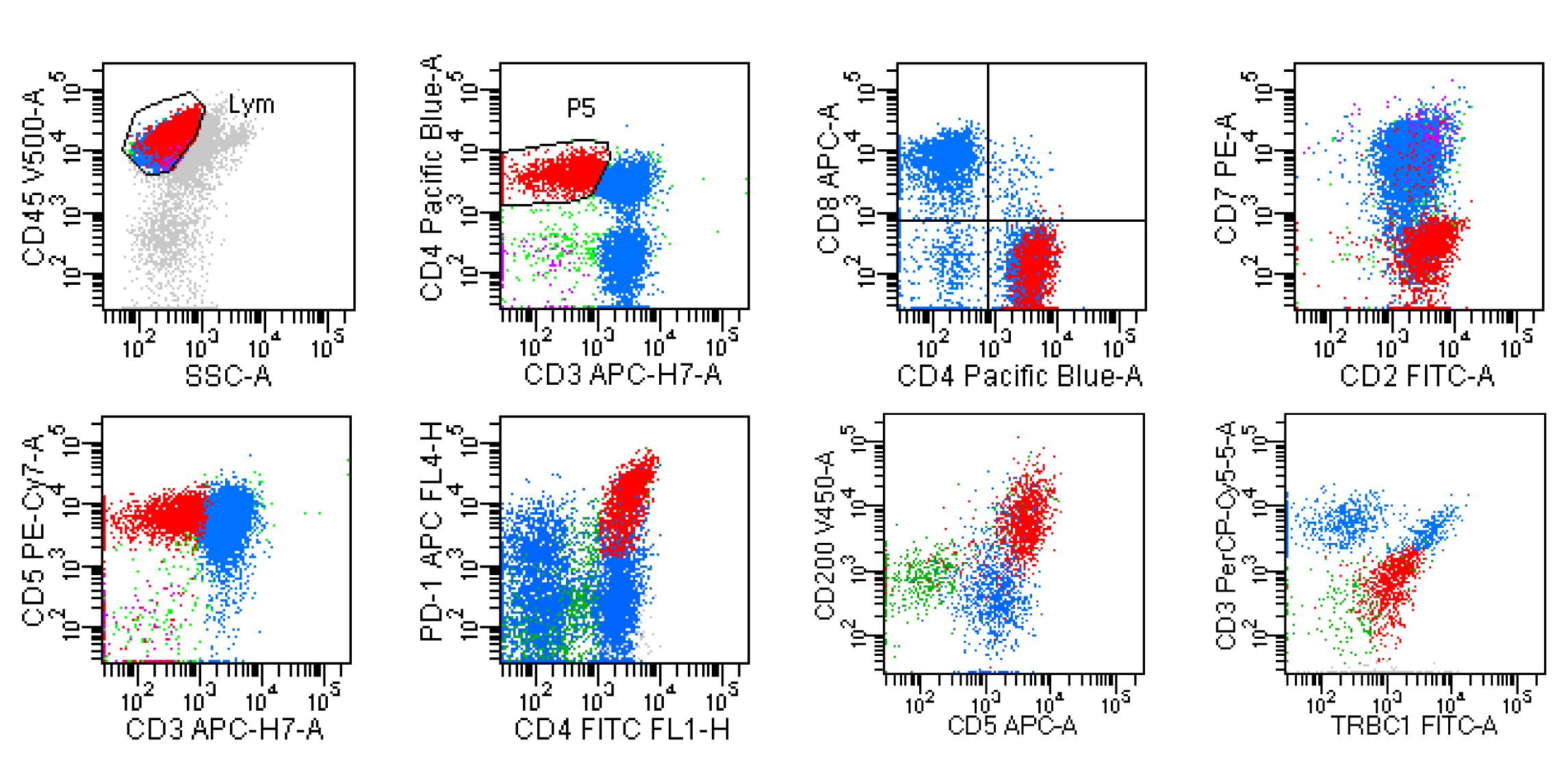

Supplement: Supplementary file 3 — High Resolution Image (TIF 8.33 MB) [file 277_2026_6930_MOESM2_ESM.tif]

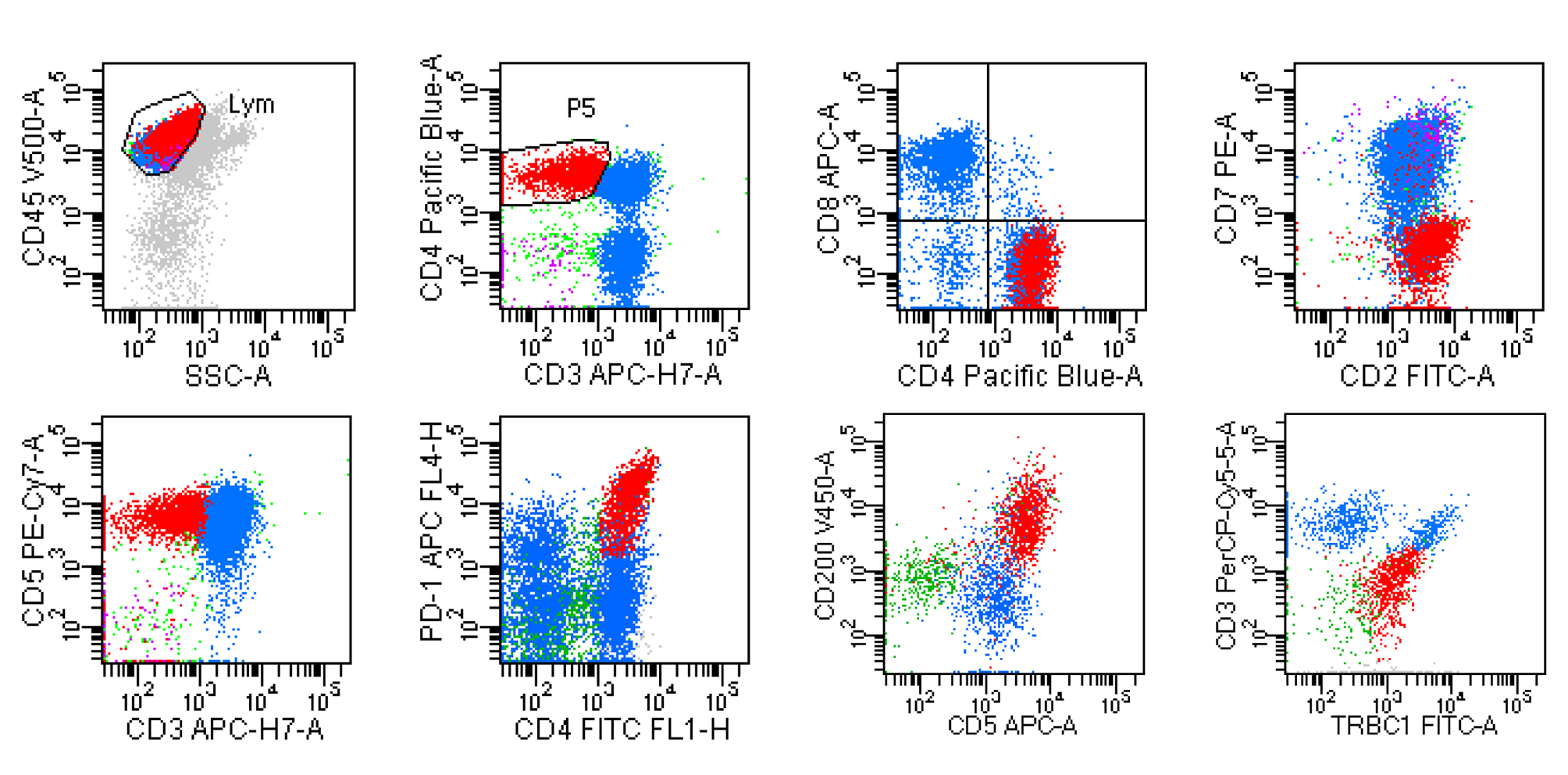

Supplement: Supplementary file 4 — Supplementary Figure 2 (PNG 455 KB) [file 277_2026_6930_Fig3_ESM.png]

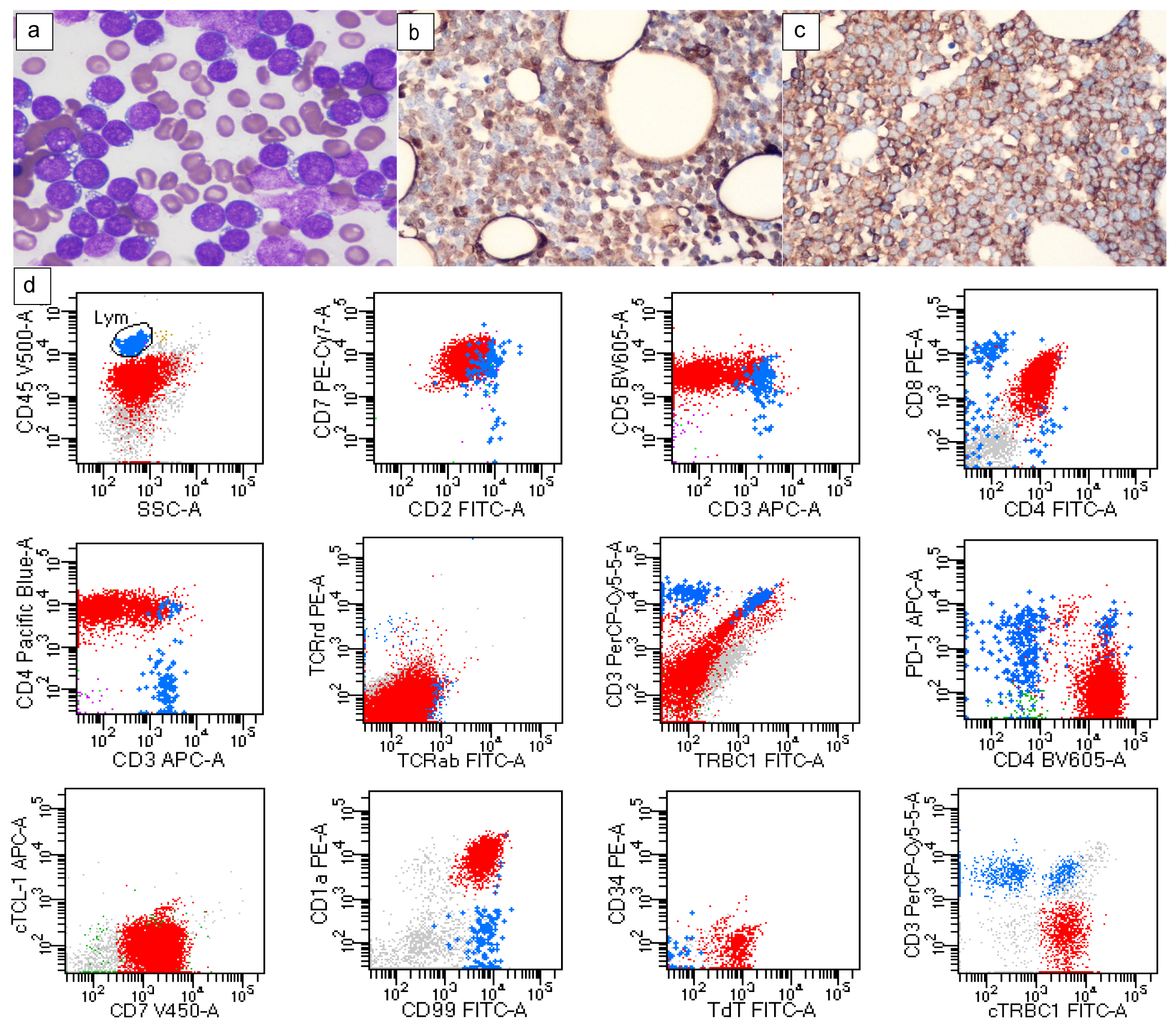

Supplement: Supplementary file 5 — High Resolution Image (TIF 17.5 MB) [file 277_2026_6930_MOESM3_ESM.tif]

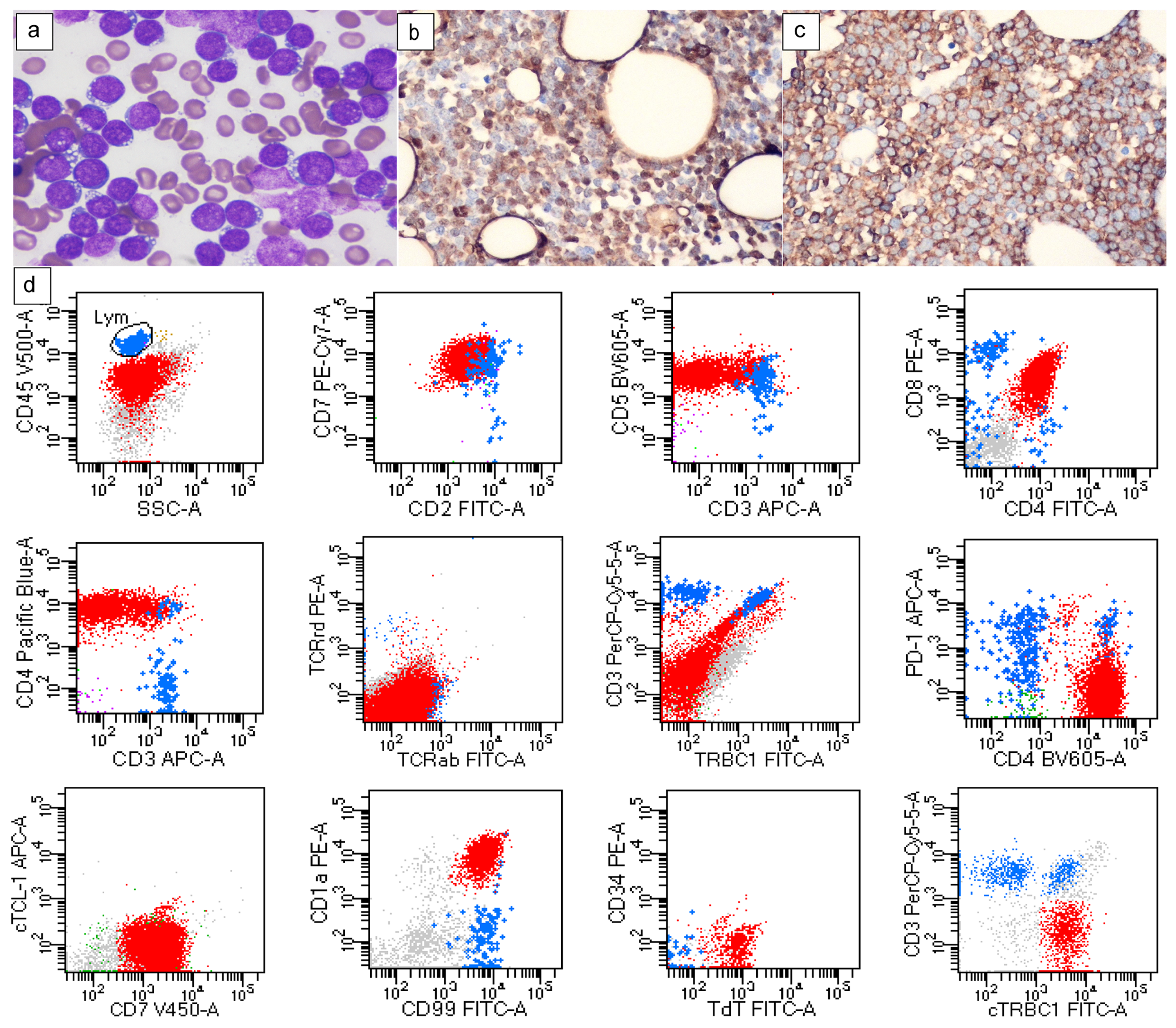

Supplement: Supplementary file 6 — Supplementary Figure 3 (PNG 2.03 MB) [file 277_2026_6930_Fig4_ESM.png]

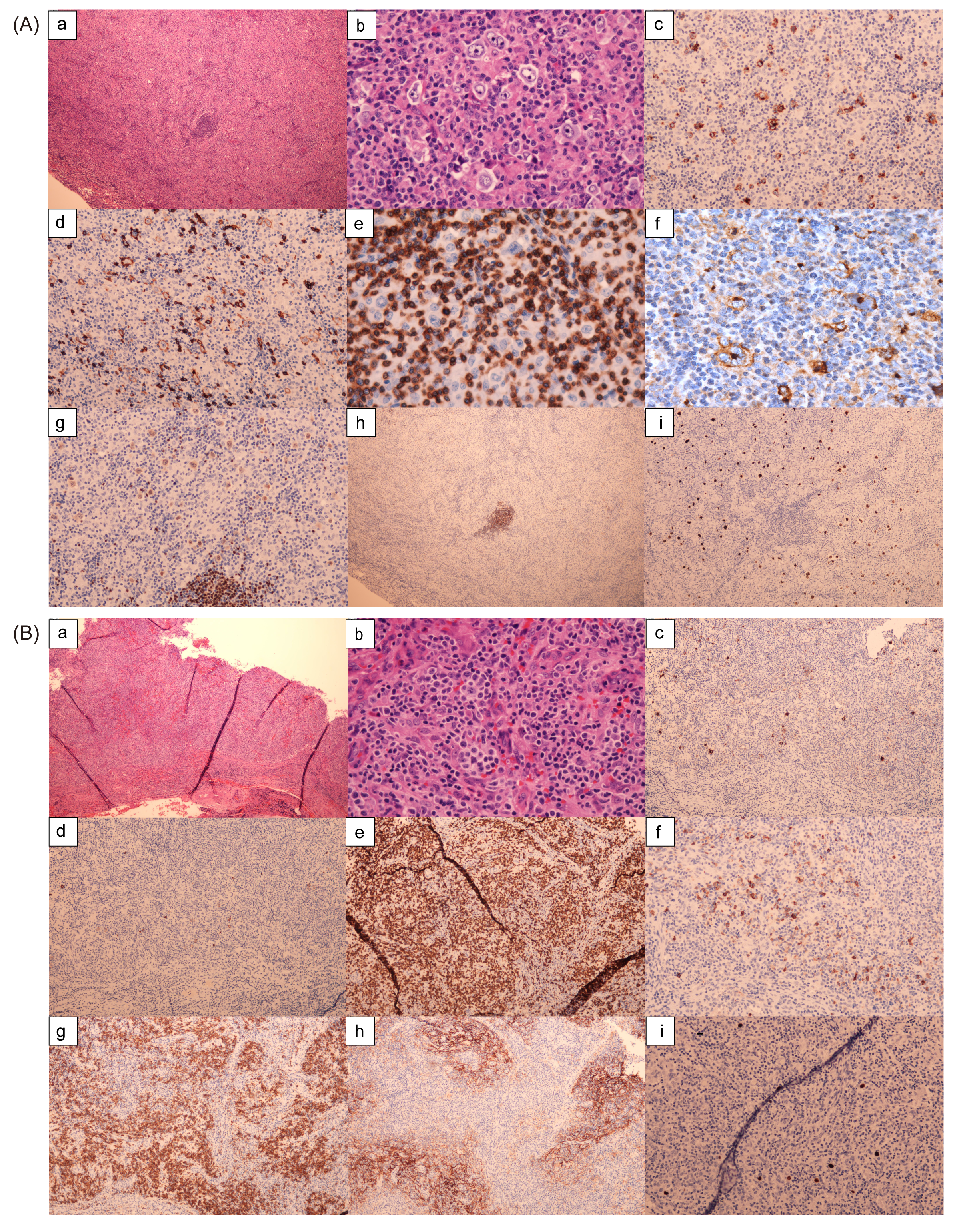

Supplement: Supplementary file 7 — High Resolution Image (TIF 40.1 MB) [file 277_2026_6930_MOESM4_ESM.tif]
